# Prediction of Severity of Acute Pancreatitis Using Total Serum Calcium and Albumin-Corrected Calcium: A Prospective Study in Tertiary Center Hospital in Nepal

**DOI:** 10.1155/2017/1869091

**Published:** 2017-12-19

**Authors:** Ashik Pokharel, Prem Raj Sigdel, Suman Phuyal, Prasan Bir Singh Kansakar, Pradeep Vaidya

**Affiliations:** Department of General and GI Surgery, Institute of Medicine, Tribhuvan University, Kirtipur, Nepal

## Abstract

**Introduction:**

Total calcium (TC) and albumin-corrected calcium (ACC) are easily accessible AP severity tests in the Primary Health Care Center of Nepal. The aim of the study was to evaluate TC and ACC as prognostic severity markers in acute pancreatitis (AP).

**Methods:**

All patients admitted in Tribhuvan University Teaching Hospital with the diagnosis of AP were studied prospectively over a period of one year from January 2015 to January 2016. TC and ACC were measured in the first 24 hours of admission in each patient. The modified Marshall score was determined at admission and at 48 hours and at any point of time during admission as per the need of the patient. Severity of acute pancreatitis was defined as per the Revised Atlanta Classification 2012.

**Results:**

80 patients of AP were included in the study. Among them, 14% were categorized as having severe AP. The mean total calcium was 8.22, 7.51, and 6.98 for mild, moderate, and severe AP, respectively, which was significant at 0.001.

**Conclusion:**

TC and ACC, measured within the first 24 hours, are useful severity predictors in acute pancreatitis.

## 1. Introduction

Acute pancreatitis (AP) is a major cause of mortality and morbidity worldwide. In approximately one-third of the patients, acute severe pancreatitis may develop, producing progressive organ dysfunction usually caused by a rapidly progressive inflammatory response, which is associated with a prolonged hospital stay and significant morbidity and mortality. Patients with mild AP have mortality rates of less than 1%, but it is rapidly increased up to 10–30% in cases with severe AP [1]. So, early detection of severe pancreatitis is essential for proper care and management and to limit its complications.

The 1992 Atlanta guidelines was the first clinically based and universally applicable classification which defined the severity and the complications of AP [2]. The Atlanta classification was revised in the year 2012, which categorizes AP into 3 states: mild, moderate, and severe based on the presence of organ failure or local complications [3]. The organ failure is determined by the modified Marshall scoring system [4].

Early detection of severe AP is important so as to deliver proper care to the patient and to avoid its complications. Total calcium and albumin-corrected calcium are simplified markers that can be readily measured and can be easily calculated and interpreted by any health persons. It has been evaluated as a mortality prognostic factor and has also been evaluated as a predictor of severe AP with infection. Albumin-corrected calcium has also been associated with severity [5].

## 2. Materials and Methods

Patients with a diagnosis of AP admitted in the surgical and medical wards of Tribhuvan University Teaching Hospital over a period of one year (January 2015–January 2016) were studied. Demographic variables including age, sex, and etiological factors were recorded. Diagnosis of acute pancreatitis, its severity, and local and systemic complications were defined as per the Revised Atlanta Classification 2012.

The diagnosis of acute pancreatitis was made if it fulfilled two of the following three features: (1) upper abdominal pain of acute onset often radiating to the back, (2) serum amylase or lipase activity greater than 3 times normal, and (3) findings on cross-sectional abdominal imaging consistent with acute pancreatitis [3]. The vitals (blood pressure, pulse, respiratory rate, and temperature) and urine output were recorded four hourly and as necessary from the time of admission until 72 hours.

Routine hematological and biochemical parameters were measured using an autoanalyzer machine available in the hospital laboratory. Serum calcium was calculated using Biotech 3000 Analyzer using cresolphthalein method, and serum albumin was calculated using bromocresol green method.

In order to evaluate TC and ACC as prognostic factors of severity, the lowest TC values were collected within the first 24 h of hospital admittance. These values were then corrected based on the serum albumin level, thus obtaining the ACC using the following formula: (ACC = TC + (0.8 ∗ [4 – serum albumin concentration])) [6].

The modified Marshall score was determined at admission and at 48 hours and at any point of time during admission as per the need of the patient [3]. Severity of AP as defined by the Revised Atlanta Classification was taken into account [3]. Ethical clearance was obtained from the Institutional Review Board. Informed consent was obtained from each patient.

### 2.1. Collection of Data

Data were collected in pro forma from all the admitted patients with the diagnosis of acute pancreatitis. Biliary etiology was confirmed as the cause of AP in patients with gall bladder stone or bile duct stone on imaging. A combination of age, sex, and laboratory markers was used in case of difficulty to predict a biliary etiology. Alcoholic etiology was defined on the basis of a history of chronic alcohol intake or recent alcohol intake in the week prior to admission while AP of other etiologies was excluded. When a diagnosis could not be made through a history, physical examination, laboratory studies, and imaging modalities, those cases were designated as idiopathic pancreatitis.

### 2.2. Statistical Analysis

One-way ANOVA and the chi-square test were employed to establish the statistical significance of the differences between groups, based on the characteristics of the analyzed variables. Statistical significance was determined with *p*<0.05. ROC curves were used in order to establish the possible cutoff values for TC and ACC. The maximum cutoff value was utilized to calculate the sensitivity (S), specificity (Sp), positive predictive value (PPV), negative predictive value (NPV), positive likelihood ratio (PLR), and negative likelihood ratio (NLR) of each criterion by means of contingency tables. All the statistical analyses were done using Statistical Package for the Social Sciences Programme v.21 (IBM SPSS).

## 3. Results

Within the study period, a total of eighty patients were included in the study. The age of the patients ranged from 19 to 87 years. AP was common in 41–50 years of age group with a mean age of 47.82 ± 15.91 years. There was no significant difference in the age of patients in each severity grade (*p* value: 0.242). Forty-six patients (58%) who presented with acute pancreatitis were female, whereas thirty-four (42%) were male. 55 (69%) of the patients belonged to the mild group, 14 (17.0%) in moderately severe group, and 11 (14.0%) in severe group.

47 (59%) patients were admitted with biliary etiology and 33 (41%) with nonbiliary etiology. Among nonbiliary patients, alcohol-induced pancreatitis was the most common (*n* = 20). In 10% of the cases, etiology could not be identified (*n* = 8). There was no significant association between etiology and severity of the disease (*p* value > 0.05). The mean duration of stay in severe pancreatitis was 14.45 ± 4.39 days.

The mean total calcium was 8.22, 7.51, and 6.98 for mild, moderate, and severe AP, respectively, which was significant at 0.001. As compared to TC, mean values of ACC were 8.15, 7.41, and 7.01 for mild, moderate, and severe AP, respectively, which were also significant at 0.002 (Table 1).

**Figure 1:**
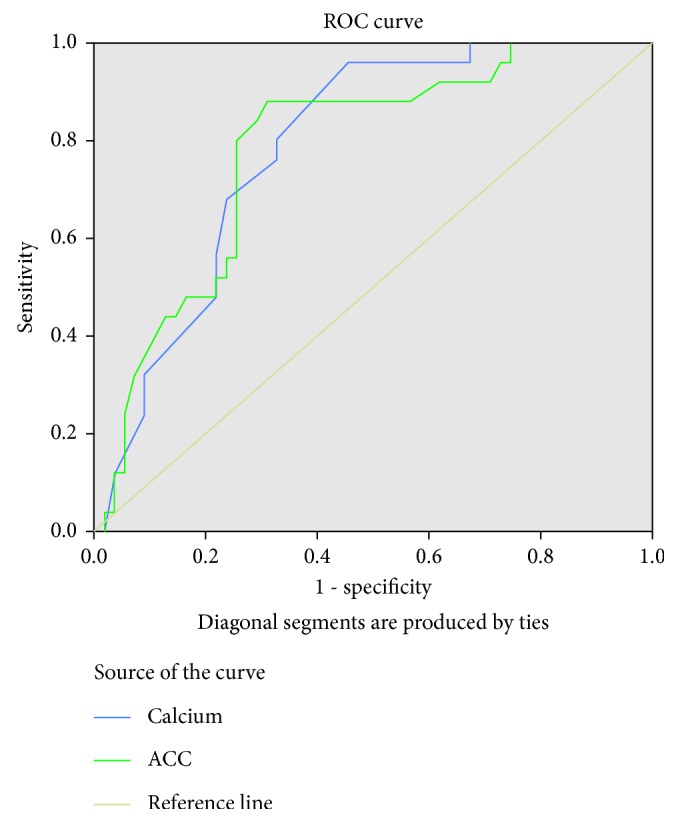
ROC (receiver-operating characteristic) curves of the total serum calcium (TC) and albumin-corrected calcium (ACC) for predicting severe AP.

A receiver-operating characteristic (ROC) analysis of the total calcium was analyzed for severe acute pancreatitis, AUC of which came out to be 0.787, which was significant (*p* value: 0.001) (Figure 1, Table 2). Similarly, ROC curve of albumin-corrected calcium analyzed for severe acute pancreatitis showed AUC of 0.781, which was also significant (*p* value: 0.002) (Figure 1, Table 2). The ROC curves of TC and ACC were comparable.

When the coordinates on the curve were analyzed (Table 3), TC of 8.20 mg/dl was computed as cutoff for severe AP with sensitivity of 96%, specificity of 54.5%, PPV of 49%, and NPV of 96.8% (Table 3). Also, when the coordinates on the curve for ACC were analyzed (Table 3), ACC of 7.72 mg/dl was computed as cutoff for severe AP with sensitivity of 88%, specificity of 69.1%, PPV of 56.4%, and NPV of 92.7% (Table 3).

## 4. Discussion

Acute pancreatitis is a common surgical emergency with mortality of severe attacks, reaching up to 30%–50% [7]. This subgroup of patients needs to be identified early in the course of the disease and needs to be aggressively treated to prevent mortality. Proper identification of the mild disease is also necessary to avoid unnecessary over treatment, thereby reducing the financial implications.

Severity assessment in acute pancreatitis was first started in 1974 by Ranson et al. [8]. The Ranson, Glasgow, and APACHE II score are few of the commonly used scoring systems [9]. Limitations of these scoring systems include delay in complete scoring where it takes 48 hours to complete Ranson and Glasgow scoring systems to complete the assessment, while APACHE II score is very cumbersome to calculate [9]. Hypocalcemia is one of the components of Ranson's scoring system done to assess the severity of pancreatitis. Ammori et al. reported that hypocalcemia was more frequent during severe attack as compared to mild attack of pancreatitis (86% versus 39%, *p*<0.001) [10]. Prevalence of hypocalcemia ranges between 15% and 88% in critically ill patients depending on the setting and cutoffs used [11, 12].

Proposed mechanisms for hypocalcemia in early phase are autodigestion of mesenteric fat by pancreatic enzymes and release of free fatty acids, which form calcium salts, transient hypoparathyroidism, and hypomagnesemia [13–15]. Later stages of pancreatitis are frequently complicated by sepsis. Whitted et al. proposed that increased circulating catecholamines in sepsis cause a shift of circulating calcium into the intracellular compartment, leading to relative hypocalcemia. This causes increased PTH secretion by negative feedback loop, leading to further increase in intracellular calcium overload, oxidative stress, and cell death [16]. Hypomagnesaemia-induced impaired PTH secretion and action, relative PTH deficiency, and vitamin D deficiency are some of the other plausible causes.

The largest multicenter study conducted in four hospitals of Australia on a cohort of 7024 patients showed that iCa < 0.8 mmol/L was an independent predictor of mortality in intensive care unit (ICU) patients [17]. In a study by Chhabra et al. [18], patients with hypocalcemia in acute pancreatitis were found to have a significantly higher frequency of persistent organ failure and need for intervention as well as mortality compared with patients with normal serum calcium levels. The low corrected serum calcium levels had a sensitivity of 81.3% and a specificity of 87.6%, whereas lower ionized serum calcium levels had a sensitivity of 81.3% and specificity of 77.5% for prediction of mortality [18].

In our study, severity of AP was not related to the age of the patient. There was no difference between sex and the severity of AP in our study. Similarly, there was no association between etiology and severity of AP. In a review article by Meher et al. [19] the total calcium and albumin-corrected calcium were considered as emerging potential biomarkers for prediction of severity in AP.

Our study showed decreasing TC and ACC for increasing severity of the disease. Although this study failed to differentiate between moderate and mild AP, cutoff of 8.20 mg/dL predicted the occurrence of severe AP with sensitivity of 96%, specificity of 54.50%, positive predictive value of 49%, and negative predictive value of 96.8%. When 7.5 mg/dl is taken as a cutoff value for the total serum calcium in our study, the sensitivity and negative predictive values decreased up to 68% and 84%, respectively, and while comparing the sensitivity, specificity, and predictive values of TC with that of the study done by Gutierrez-Jimenez et al. [5], our sensitivity was much higher (96% versus 67%) and a positive predictive value (49% versus 27%) was obtained when the cutoff value was taken as 8.20 mg/dl. The higher sensitivity and positive predictive value could be due to the higher cutoff value in our study. The higher cutoff value for TC in our study could be due to a higher normal range of calcium in our lab which is in the range of 8.4–10.4 mg/dl. But the exact reason could not be found.

Similarly, when ACC of 7.72 mg/dl was computed as cutoff for severe AP, we found sensitivity of 88%, specificity of 69.1%, PPV of 56.4%, and NPV of 92.7%. When 7.5 mg/dl is taken as a cutoff value for albumin-corrected calcium in our study, the sensitivity and negative predictive values decreased up to 80% and 89.1%, respectively, and while comparing the sensitivity, specificity, and predictive values of ACC with that of the study done by Gutierrez-Jimenez et al. [5], our sensitivity was much higher (88% versus 67%) and a positive predictive value (56.4% versus 40%) was obtained when the cutoff value was taken as 7.72 mg/dl. As explained above, the higher sensitivity and PPV could be due to the higher cutoff value in our study. The higher cutoff value for ACC in our study could be due to a higher normal range of calcium in our lab which is in the range of 8.4–10.4 mg/dl.

When we compare the sensitivity, specificity, and predictive values of TC and ACC, TC seems to better predict the severity of acute pancreatitis as the value of ACC varied with various other parameters including the nutritional status and chronic liver disease, and it also takes a little time for albumin to get depleted in diseases.

A large number of health care centers have access to TC and ACC use, but not as many have access to the resources required for using the APACHE-II scale and other AP severity markers, such as C-reactive protein, interleukin 6, or procalcitonin. Serum calcium and albumin for calculating ACC are simple biochemical markers that are routinely determined in the majority of hospital centers. Their use as prognostic factors of severity in AP would be valuable for identifying those persons who require intensive care, even at the primary and secondary care center levels.

## 5. Conclusion

Serum calcium and albumin-corrected calcium obtained within the first 24 hours of hospital admission are useful predictors of severity in acute pancreatitis. This will not replace the currently accepted scoring systems, but these are simplified markers that can be readily measured and can be easily calculated and interpreted by any health staff. With an adequate interpretation of their cutoff points, they can be used routinely in every case of acute pancreatitis to assess its severity, predict complications, and identify the patients who require intensive care support even in primary and secondary care centers.

## Figures and Tables

**Table 1:** Comparison of the mean total serum calcium and albumin-corrected calcium as per severity.

Factor	Total population (*n* = 80)	Mild acute pancreatitis (*n* = 55)	Moderately severe pancreatitis (*n* = 14)	Severe acute pancreatitis (*n* = 11)	*p* value
Total serum calcium (mg/dl), mean ± SD	7.92 ± 1.09	8.22 ± 1.11	7.51 ± 0.61	6.98 ± 0.67	0.001
Albumin-corrected calcium (mg/dl), mean ± SD	7.87 ± 1.07	8.15 ± 1.08	7.41 ± 0.84	7.01 ± 0.42	0.002

**Table 2:** Table showing area under the curve (AUC) and significance (severe AP).

				95% CI	
Test variable	Area	Std. error	*p* value	Lower bound	Upper bound
Total calcium	0.787	0.050	0.001	0.689	0.886
ACC	0.781	0.054	0.001	0.676	0.887

**Table 3:** Sensitivity, specificity, predictive values, and likelihood ratios of TC and ACC for predicting progression to severe acute pancreatitis.

Factor	S (%)	Sp (%)	PPV (%)	NPV (%)	LR+	LR−
Total calcium (≤8.2 mg/dl)	96.0	54.50	49.0	96.8	2.11	0.07
Albumin-corrected calcium (≤7.72 mg/dl)	88.0	69.10	56.4	92.7	2.85	0.17

*Odds ratio (OR)* for patients with calcium value ≤ 8.2 mg/dl is found to be 28.8 (*p* value = 0.01) (95% CI: 3.6–228.1) and OR for patients with ACC value ≤ 7.72 mg/dl is found to be 16.4 (*p* value = 0.001) (95% CI: 4.3–62.3).
